# The Pathogen-Occupied Vacuoles of *Anaplasma phagocytophilum* and *Anaplasma marginale* Interact with the Endoplasmic Reticulum

**DOI:** 10.3389/fcimb.2016.00022

**Published:** 2016-03-01

**Authors:** Hilary K. Truchan, Chelsea L. Cockburn, Kathryn S. Hebert, Forgivemore Magunda, Susan M. Noh, Jason A. Carlyon

**Affiliations:** ^1^Department of Microbiology and Immunology, Virginia Commonwealth University School of MedicineRichmond, VA, USA; ^2^Program in Vector Borne Diseases, Department of Veterinary Microbiology and Pathology, Washington State UniversityPullman, WA, USA; ^3^The Paul G. Allen School for Global Animal Health, Washington State UniversityPullman, WA, USA; ^4^Animal Disease Research Unit, Agricultural Research Service, U. S. Department of AgriculturePullman, WA, USA

**Keywords:** *Anaplasmataceae*, *Rickettsia*, intracellular bacteria, endoplasmic reticulum, Rab, pathogen synapse

## Abstract

The genus *Anaplasma* consists of tick-transmitted obligate intracellular bacteria that invade white or red blood cells to cause debilitating and potentially fatal infections. *A. phagocytophilum*, a human and veterinary pathogen, infects neutrophils to cause granulocytic anaplasmosis. *A. marginale* invades bovine erythrocytes. Evidence suggests that both species may also infect endothelial cells *in vivo*. In mammalian and arthropod host cells, *A. phagocytophilum* and *A. marginale* reside in host cell derived pathogen-occupied vacuoles (POVs). While it was recently demonstrated that the *A. phagocytophilum*-occupied vacuole (ApV) intercepts membrane traffic from the *trans*-Golgi network, it is unclear if it or the *A. marginale*-occupied vacuole (AmV) interacts with other secretory organelles. Here, we demonstrate that the ApV and AmV extensively interact with the host endoplasmic reticulum (ER) in endothelial, myeloid, and/or tick cells. ER lumen markers, calreticulin, and protein disulfide isomerase, and the ER membrane marker, derlin-1, were pronouncedly recruited to the peripheries of both POVs. ApV association with the ER initiated early and continued throughout the infection cycle. Both the ApV and AmV interacted with the rough ER and smooth ER. However, only derlin-1-positive rough ER derived vesicles were delivered into the ApV lumen where they localized with intravacuolar bacteria. Transmission electron microscopy identified multiple ER-POV membrane contact sites on the cytosolic faces of both species' vacuoles that corresponded to areas on the vacuoles' lumenal faces where intravacuolar *Anaplasma* organisms closely associated. *A. phagocytophilum* is known to hijack Rab10, a GTPase that regulates ER dynamics and morphology. Yet, ApV-ER interactions were unhindered in cells in which Rab10 had been knocked down, demonstrating that the GTPase is dispensable for the bacterium to parasitize the ER. These data establish the ApV and AmV as pathogen-host interfaces that directly engage the ER in vertebrate and invertebrate host cells and evidence the conservation of ER parasitism between two *Anaplasma* species.

## Introduction

*Anaplasma phagocytophilum* and *Anaplasma marginale* are tick-transmitted obligate intracellular bacterial pathogens of the Family *Anaplasmataceae* that cause debilitating and potentially fatal diseases (Carlyon, 2012). *A. phagocytophilum* infects neutrophils to cause granulocytic anaplasmosis in humans and animals (Truchan et al., 2013). Over the decade leading up to 2012, the most recent year for which United States Centers for Disease Control (CDC) statistics are available, the number of human granulocytic anaplasmosis (HGA) cases reported annually to the CDC rose nearly seven-fold (CDC, 2013). The disease also continues to emerge in Europe and Asia (Truchan et al., 2013). HGA is an acute febrile illness that can be accompanied by non-specific symptoms including headache, malaise, myalgia, elevated liver enzymes, leukopenia, and thrombocytopenia (Truchan et al., 2013). *A. marginale* is a strict bovine pathogen that is endemic throughout the southern Atlantic, Gulf coast, and several Midwestern and Western U. S. states, as well as Mexico, Central and South America, and the Caribbean Islands. It infects erythrocytes, which can result in anemia, weight loss, reduced growth, and milk production, and abortion in pregnant cattle (Kocan et al., 2010; Suarez and Noh, 2011). Following resolution of acute disease, bovine anaplasmosis can remain chronic for the life of the animal and is estimated to cost the U.S. and South American cattle industries hundreds of millions of dollars each year (Kocan et al., 2003; Suarez and Noh, 2011).

*A. phagocytophilum* and *A. marginale* replicate within host cell-derived vacuoles (Carlyon, 2012). Both will infect mammalian and tick cell lines, including human promyelocytic HL-60 cells (*A. phagocytophilum* only), *Ixodes scapularis* embryonic ISE6 cells (both *Anaplasma* spp.), and primate RF/6A endothelial cells (both species) (Goodman et al., 1996; Woldehiwet et al., 2002; Munderloh et al., 2004; Zivkovic et al., 2009). RF/6A cells are particularly useful models for studying the cellular microbiology of these bacteria because they are large and flat, making them ideal for imaging (Munderloh et al., 2004; Sukumaran et al., 2011; Beyer et al., 2014; Truchan et al., 2016). Moreover, *A. phagocytophilum* and *A. marginale* have been detected in endothelial cells of tissue sections recovered from experimentally infected animals (Herron et al., 2005; Wamsley et al., 2011). During growth in tissue culture cells, both *Anaplasma* spp. cycle between a dense-cored (DC) morphotype that binds and invades host cells and a reticulate cell (RC) morphotype that replicates inside the pathogen-occupied vacuole (POV) (Munderloh et al., 2004; Troese and Carlyon, 2009). Like many professional vacuolar bacterial pathogens (Brumell and Scidmore, 2007; Sherwood and Roy, 2013), *A. phagocytophilum* selectively recruits a subset of Rab GTPases to its vacuole (Huang et al., 2010a). We recently reported that one such GTPase, Rab10, is critical for the pathogen to parasitize exocytic traffic from the *trans*-Golgi network (Truchan et al., 2016). Whether the *A. phagocytophilum*-occupied vacuole (ApV) or *A. marginale*-occupied vacuole (AmV) hijacks other arms of the secretory pathway in mammalian or tick host cells is unknown.

Nascent proteins that are destined for either secretion or for the plasma membrane, secretory, or endocytic organelles are first translocated into the endoplasmic reticulum (ER) where they are processed and subjected to quality control. Once thought of as a single large organelle, the ER is actually an assemblage of several membrane domains—the ribosome studded rough ER (RER), smooth ER (SER), mitochondria associated membrane (MAM), ER exit sites (ERESs), and ER quality control compartment (ERQC) (Lynes and Simmen, 2011; Benyair et al., 2015). Proteins are synthesized, N-terminally glycosylated, and translocated into the RER. Here, the glycoproteins are initially processed and begin the quality control process wherein they are cycled among the RER, SER, MAM, and ERQC. Properly folded secretory glycoproteins are segregated into ERESs, transported to the ERGIC (ER-to-Golgi intermediate compartment) and subsequently to the Golgi. In the ERQC, terminally misfolded glycoproteins are targeted for ER associated degradation (ERAD), a process by which the misfolded proteins are retrotranslocated to the cytosol, where they are degraded by the ubiquitin proteasome system (Benyair et al., 2015).

Rab10, which is important for *A. phagocytophilum* TGN parasitism (Truchan et al., 2016), not only directs exocytic traffic from the TGN (Liu and Storrie, 2012) but also regulates ER dynamics and morphology (English and Voeltz, 2013). Moreover, Rab1, a GTPase that directs vesicular traffic from the ER to the Golgi apparatus (Stenmark, 2009), is also recruited to the ApV (Huang et al., 2010a). Given these phenomena, the paucity of information on AmV-host cell interactions, and the dearth of knowledge on the cellular microbiology of *Anaplasma* spp. in tick cells, we investigated if the ApV and AmV engage the ER during infection of mammalian and tick host cells. Our data reveal that both POVs interact with the ER and that derlin-1-positive vesicles are delivered into their lumen. Thus, the ability to hijack the secretory pathway is conserved between *A. phagocytophilum* and *A. marginale*.

## Materials and methods

### Cultivation of uninfected and *Anaplasma* spp. infected cell lines

Uninfected and *A. phagocytophilum* (NCH-1 strain)-infected human promyelocytic HL-60 cells (CCL-240; American Type Culture Collections [ATCC, Manassas, VA]), RF/6A rhesus monkey choroidal endothelial cells (CRL-1780, ATCC), and ISE6 cells were cultured as described (Huang et al., 2010a, 2012; Beyer et al., 2014). *A. marginale* (St. Maries strain)-infected RF/6A cells and uninfected and *A. marginale* infected ISE6 cells were gifts from Ulrike Munderloh (University of Minnesota, Minneapolis, MN). *A. marginale* infected ISE6 cells were cultured identically to *A. phagocytophilum* infected ISE6 cells. *A. marginale* infected RF/6A cells were maintained in 25 cm^2^ cell culture flasks as follows. When ≥ 80% of the cells had lysed and the media contained infectious *A. marginale* organisms, 5 ml of the bacteria laden media was transferred to a 25 cm^2^ cell culture flask containing naïve RF/6A cells that were nearly confluent. Uninfected RF/6A cells were grown to near confluency in 25 cm^2^ cell culture flasks. Human embryonic kidney HEK-293T cells were cultured in Dulbecco's Modified Eagle's Medium with L-Glutamine, 4.5 g/L D-Glucose, and 100 mg/L sodium pyruvate (DMEM; Invitrogen, Carlsbad, CA) supplemented with 10% fetal bovine serum (FBS), 1X MEM Non-Essential Amino Acids (Invitrogen), and 15 mM HEPES (4-(2-hydroxyethyl)-1-piperazineethanesulfonic acid) (Affymetrix, Cleveland, OH) at 37°C with 5% CO_2_.

### Immunofluorescence microscopy and western blot

Cells for immunofluorescence assays were grown and infected on #1½12 × 12-mm glass coverslips for laser-scanning confocal microscopy (LSCM; Electron Microscopy Sciences, Hatfield, PA) or #1½HP (i.e., 0.17 ± 0.005 mm thick) high performance glass coverslips (Zeiss, Thornwood, NY) for structured illumination microscopy (SIM). The cells were fixed in 4% paraformaldehyde (Electron Microscopy Sciences) for 30 min followed by permeabilization with 0.5% Triton X-100 for 10 min. Immunofluorescence labeling was performed as previously described (Beyer et al., 2014). For LCSM, coverslips were mounted with Prolong Gold Anti-fade reagent with DAPI (4′,6-diamidino-2-phenylindole, Invitrogen) and images were obtained using a Zeiss LSM 700 laser-scanning confocal microscope. Three-dimensional rendering and movies were generated using Volocity Image Analysis Software (PerkinElmer, Waltham, MA). For SIM, coverslips were stained with 1 μg/mL DAPI in 1X PBS for 5 min and mounted with Prolong Gold Anti-fade reagent lacking DAPI (Invitrogen). Images were obtained using a Nikon N-SIM super resolution microscope. Lysates of HL-60 cells, host cell-free *A. phagocytophilum* organisms, or gradient centrifugation fractions were analyzed by SDS-PAGE and Western blot as described (Troese et al., 2011). Primary antibodies used for immunofluorescence and Western blot analyses targeted calreticulin (Sigma-Aldrich, St. Louis, MO), derlin-1 (Sigma-Aldrich and Santa Cruz Biotechnologies [Santa Cruz, CA])), protein disulfide-isomerase (PDI; Sigma-Aldrich), kinectin-1 (Sigma-Aldrich), reticulon-4 (LifeSpan Biosciences Inc, Seattle, WA), *A. phagocytophilum* APH0032 (Huang et al., 2010b), *A. phagocytophilum* P44 (Huang et al., 2010b), and *A. marginale* major surface protein 5 (Msp5) (Visser et al., 1992) (monoclonal antibody clone AnaF16c1, kindly provided by Beverly Hunter and Guy Palmer, Washington State University, Pullman, WA). Alexa Fluor fluorochrome- or horseradish peroxidase-conjugated secondary antibodies were obtained from Invitrogen or Cell Signaling, respectively.

### Infection assays

HL-60 cells were infected with *A. phagocytophilum* DC organisms released from infected HL-60 cells by sonication as described (Seidman et al., 2015). ISE6 cells were infected with *A. phagocytophilum* and *A. marginale* as described for *A. phagocytophilum* (Huang et al., 2012). RF/6A cells were infected with *A. phagocytophilum* and *A. marginale* organisms that had been naturally released from infected RF/6A cells into the culture media as follows. Adherent host cells to be infected were seeded onto #1½12 × 12-mm glass coverslips (Electron Microscopy Sciences) and overlaid with 200 μl of *A. phagocytophilum* or *A. marginale* laden media from heavily infected RF/6A cells. Twenty four-well plates containing the coverslips and bacteria laden media were centrifuged at 1000 *g* for 3 min to spin the bacteria onto the host cell surfaces followed by a 1 h incubation at 37°C with 5% CO_2_. The cells were washed with 1X PBS to remove unbound bacteria and fresh media was added. The cells were returned to 37°C with 5% CO_2_ for various time periods, after which they were fixed in 4% PFA and examined using immunofluorescence microscopy. To assess the effect of ectopic overexpression of mCherry-derlin-1 on the *A. phagocytophilum* load, HEK-293T cells were transfected with plasmids to enable expression of mCherry-derlin-1 (Nery et al., 2011) (a kind gift from Xandra O. Breakefield [Harvard Medical School, Boston, MA] and Iona A. Armata [Florida State University, Tallahassee, FL]), mCherry (mCherry2-C1 plasmid #54563; originally from Michael Davidson; Addgene, Cambridge, MA) or mock-transfected as previously described (VieBrock et al., 2014) for 6 h followed by incubation with host cell-free *A. phagocytophilum* organisms for 8 or 24 h prior to processing for LSCM analysis. Alternatively, HEK-293T cells were first infected with *A. phagocytophilum* or mock infected, transfected for 6 h, and processed for LSCM analysis.

### Density gradient centrifugation

2 × 10^7^ uninfected or infected HL-60 cells were washed with ice-cold 1x PBS twice followed by one wash in cold homogenization buffer (250 mM sucrose, 10 mM Tris-HCl, pH 7.4,1 mM EDTA). The cells were suspended in 1 mL ice-cold homogenization buffer with protease inhibitors (Roche, Indianapolis, IN) and homogenized in a type B dounce homogenizer (Gerresheimer Kimble Chase LLC, Vineland, NJ) for approximately 30 strokes until ≥ 90% of the host cells were lysed, as verified by the trypan blue exclusion assay. The homogenate was centrifuged at 500 *g* for 5 min to remove nuclei and unbroken cells. The post-nuclear supernatant was overlaid on a 5, 15, 25% continuous Opti-prep (Sigma-Aldrich) gradient and centrifuged at 200,000 *g* for 3 h in an Optima XE-100 ultracentrifuge (Beckman Coulter, Indianapolis, IN). Twelve 1-ml fractions were collected and concentrated by trichloroacetic acid precipitation. Equal volumes of fractions 1–9 were Western blotted and screened with calreticulin and P44 antibodies.

### siRNA knock down

4 × 10^5^ HEK-293 cells were seeded onto #1½12 × 12-mm glass coverslips (Electron Microscopy Sciences). After 16–20 h, 80 ul of 5 uM ON-TARGETplus human Rab10 or derlin-1 siRNA SMARTpool or non-targeting siRNA (GE Dharmacon, Lafayette, CO) was mixed with 320 ul of media and added to the wells. After 72 h, 200 ul of media containing *A. phagocytophilum* organisms that had been released from infected RF/6A cells was added and the bacteria were spun onto the cells as described above. At 24 or 48 h post-infection, cells were harvested for Western blot analysis to confirm knockdown, processed for microscopy analyses, or processed for quantitative PCR (QPCR) analyses as described previously (Truchan et al., 2016). Statistical significance (*P* < 0.05) was evaluated using the Prism 5.0 software package (Graphpad, San Diego, CA).

## Results

### *A. phagocytophilum*-occupied vacuoles (ApVs) interact with the host ER

**Figure 1:**
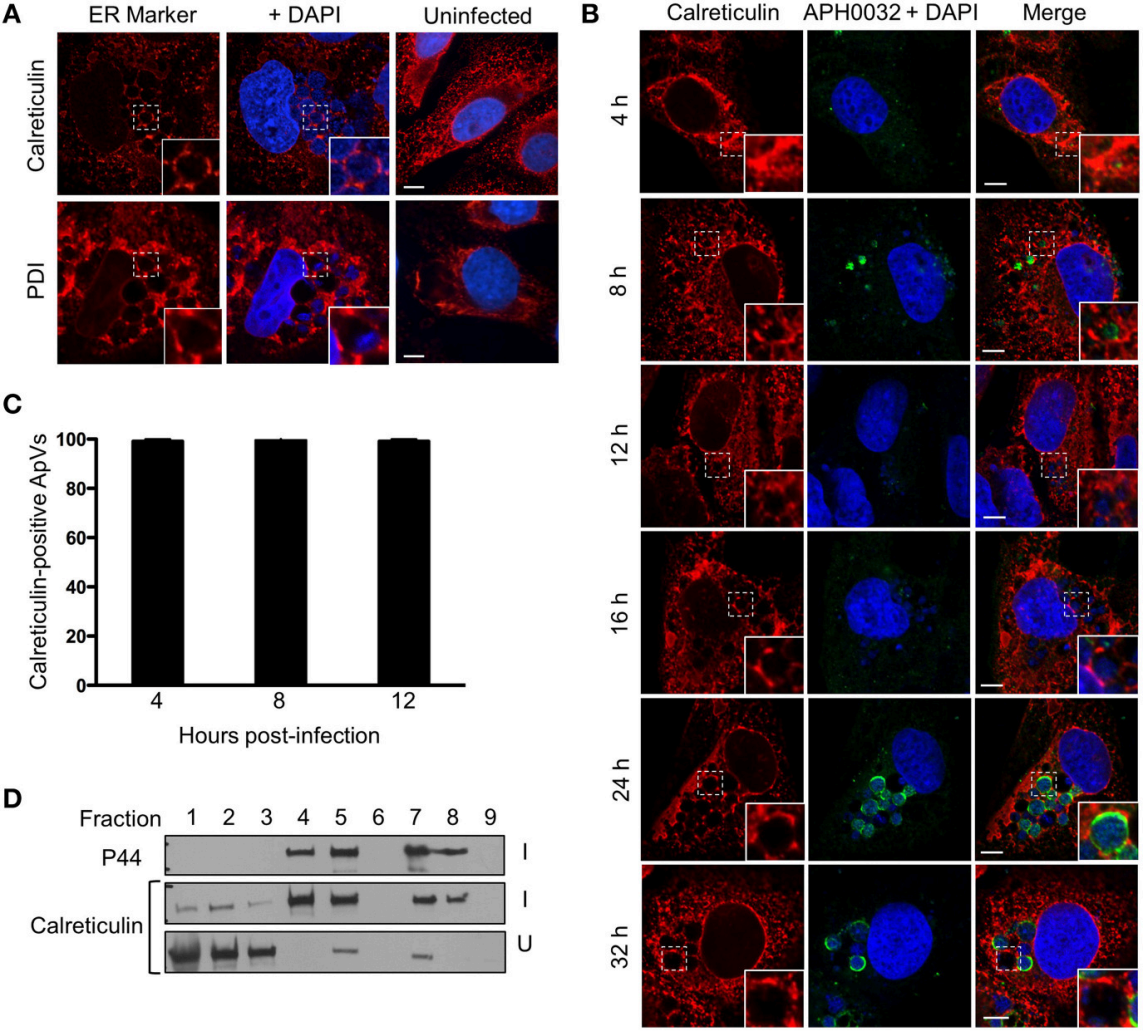
**The ApV engages the host ER throughout *A. phagocytophilum* infection of mammalian host cells. (A)** ER markers localize to and within the ApV in mammalian cells. *A. phagocytophilum* infected RF/6A cells that had been screened with antibodies against calreticulin or PDI were visualized using LSCM. **(B)** The ER is recruited to the ApV early and the association is retained throughout the course of infection. RF/6A cells that had been synchronously infected with *A. phagocytophilum* organisms were screened with antibodies against calreticulin and the pathogen derived ApV membrane protein, APH0032, and examined at several post-infection time points using LSCM. **(A,B)** Host cell nuclei and bacterial DNA were stained with DAPI (blue). The regions that are demarcated by hatched lined boxes indicate the regions magnified in the insets that are demarcated by solid lined boxes. Scale bars, 5 μm. **(C)** Percentages of calreticulin-positive ApVs, as identified by DAPI stained intravacuolar *A. phagocytophilum* bacteria, over the course of a synchronous infection. Data are the means and standard deviations for triplicate samples. **(D)** Calreticulin cofractionates with *A. phagocytophilum* organisms. Uninfected (U) or *A. phagocytophilum*-infected HL-60 cells (I) were homogenized and the post-nuclear supernatants were separated by density gradient centrifugation. Successive one-ml fractions were analyzed by Western blot using antibodies against calreticulin and the *A. phagocytophilum* major surface protein, P44. Results shown are representative of two experiments with similar results.

To investigate if ApVs engage the host ER, *A. phagocytophilum* infected RF/6A cells were fixed, screened with antibodies specific for the ER lumen markers, calreticulin and PDI (Benham, 2012), and examined by LSCM. Both antibodies revealed a characteristic ER network-like pattern in uninfected and infected cells (Figure 1A). Calreticulin and PDI were considerably enriched in patch-like patterns at the peripheries of ApVs. To determine when ApV-ER association occurs, RF/6A cells were synchronously infected and examined at multiple post-infection time points using antibodies against calreticulin and APH0032, an *A. phagocytophilum* vacuolar membrane marker that the bacterium expresses predominantly late during the infection cycle (Huang et al., 2010b). As expected (Huang et al., 2010b), APH0032-positive vacuoles were most abundantly detected at 24 and 32 h (Figure 1B), validating that the infection cycle had proceeded normally. For time points at which APH0032 was not detectable, ApVs were readily visualized due to the presence of DAPI stained bacteria within them. Calreticulin accumulated around all ApVs beginning at 4 h and remained associated throughout the remainder of the time course (Figures 1B,C). To assess for ApV-ER interactions in another mammalian host cell line, uninfected or *A. phagocytophilum* infected HL-60 cells were fractionated by a continuous density gradient fractionation method that keeps the ApV intact (Niu et al., 2012). Western blot analysis revealed altered distribution of calreticulin in the fractions of infected vs. uninfected cells, as the ER marker pronouncedly co-migrated to fractions in which the *A. phagocytophilum* outer membrane protein, P44 (Truchan et al., 2013), was most abundant (Figure 1D). This observation was similar to our previously reported finding that the *trans*-Golgi marker, TGN46, but not *cis*-Golgi marker, GM130, is specifically redistributed to ApV containing fractions. Together, these data demonstrate that the ApV associates with the host ER early and maintains this association throughout the infection cycle.

### Derlin-1-positive ER derived vesicles are delivered into the ApV lumen where they associate with *A. phagocytophilum* organisms

**Figure 2:**
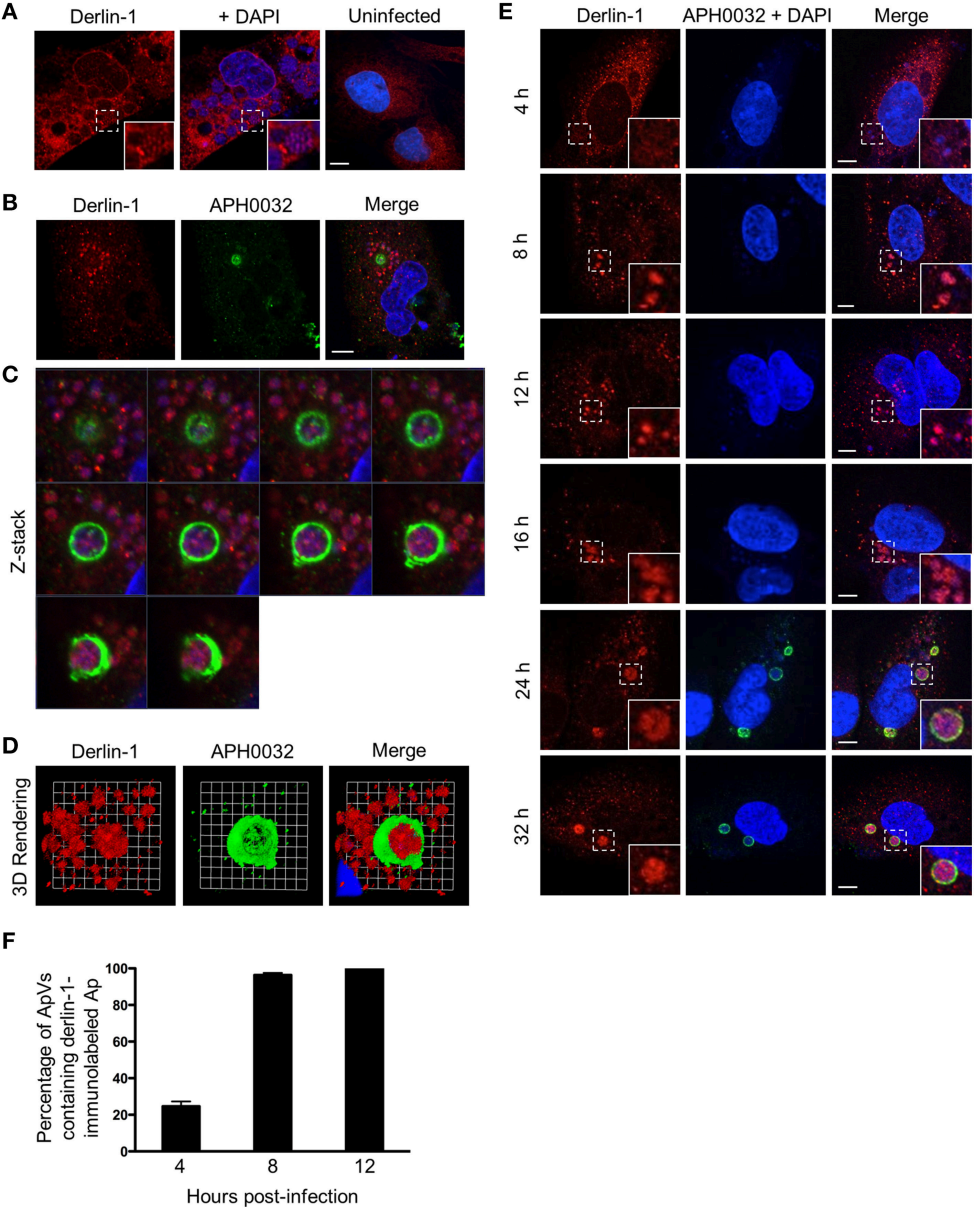
**Derlin-1-positive ER vesicles are delivered into the ApV lumen where they associate with *A. phagocytophilum* organisms. (A–D)** Derlin-1-positive vesicles are present within the ApV. *A. phagocytophilum* infected RF/6A cells were screened with antibodies against derlin-1 **(A)** or derlin-1 and APH0032 **(B)** and visualized using LSCM. **(C)** Z-stack series shows derlin-1-labeled vesicles within the ApV. Successive focal planes of the region in **(B)** that is demarcated by a hatched line box are presented. **(D)** 3D rendering of the Z-stack series presented in **(C)** shows derlin-1-positive vesicles encasing *A. phagocytophilum* organisms within the vacuole. **(E)** Derlin-1-positive vesicles are recruited to and delivered into the ApV early and continue to detected within the ApV throughout the course of infection. RF/6A cells that had been synchronously infected with *A. phagocytophilum* organisms were screened with antibodies against derlin-1 and APH0032 and visualized at several post-infection time points using LSCM. **(A,E)** Regions that are demarcated by hatched lined boxes correspond to the regions magnified in the insets that are demarcated by solid lined boxes. **(A–E)** Host cell nuclei and bacterial DNA were stained with DAPI (blue). Scale bars, 5 μm. **(F)** Percentages of ApVs in which derlin-1 signal is detectable within the lumen closely associated with intravacuolar *A. phagocytophilum* organisms over the course of a synchronous infection. Data are the means and standard deviations for triplicate samples. Results in all panels are representative of two experiments with similar results.

Given that two ER lumen markers accumulate around the ApV, it was next assessed if the ER membrane associated protein, derlin-1, exhibited a similar recruitment pattern. Derlin-1 is an ER resident protein that is recruited to the ERQC to form part of a membrane-associated complex that directs terminally misfolded proteins to the cytosol for ERAD (Benyair et al., 2015). Derlin-1 displayed a vesicular labeling pattern that, analogous to that observed for calreticulin and PDI, accumulated around ApV peripheries (Figures 2A,B). Notably, derlin-1 signal was also detected within ApVs in close proximity to DAPI-stained *A. phagocytophilum* organisms. To more closely examine the possible delivery of derlin-1 positive vesicles into the ApV lumen, a representative APH0032-positive ApV was subjected to z-section image analysis and three-dimensional (3D) rendering. Derlin-1-positive vesicles were observed in close proximity to intravacuolar bacteria within the ApV throughout the stack of z-section images (Figures 2C,D; Supplementary Movie 1). The timing of derlin-1-positive vesicle localization to and within ApVs was similar to that of calreticulin and PDI immunolabeling of the cytosolic face of the ApV (Figure 1B), as both were modest at 4 h and considerably more pronounced at all subsequent time points examined (Figures 2E,F).

### Derlin-1 immunolabeling of intravacuolar *A. phagocytophilum* bacteria is specific and is reproducible among different derlin-1 antibodies

**Figure 3:**
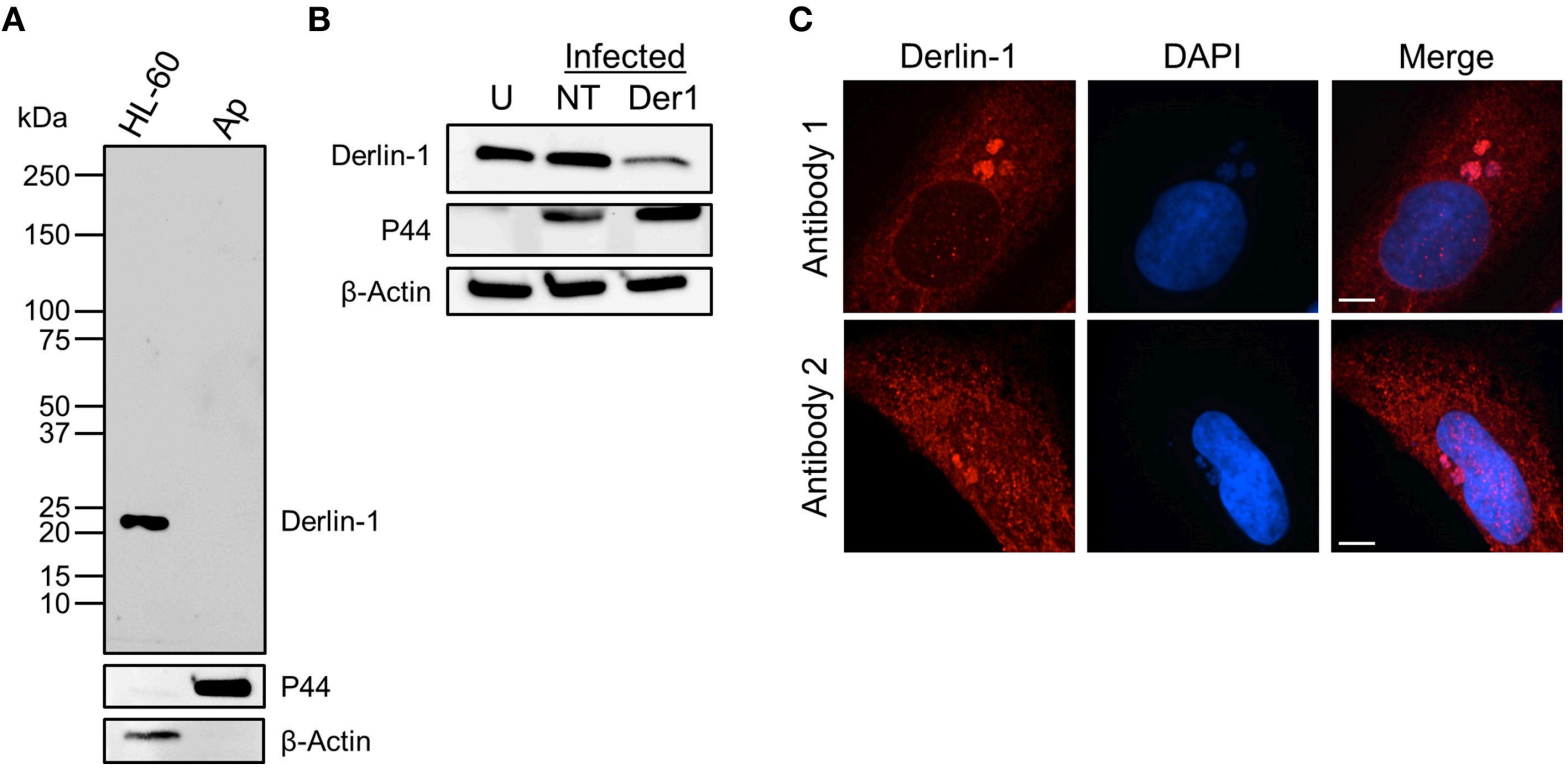
**Derlin-1 immunolabeling of intravacuolar *A. phagocytophilum* bacteria is specific and is reproducible among different derlin-1 antibodies. (A,B)** Derlin-1 antibody does not cross-react with any *A. phagocytophilum* protein. Western blotted lysates of uninfected HL-60 cells and host cell-free *A. phagocytophilum* (Ap) bacteria **(A)** or *A. phagocytophilum* infected derlin-1 siRNA treated (Der1), non-targeting siRNA treated (NT), or uninfected, untreated (U) control HEK-293T cells **(B)** were probed with antibodies against derlin-1, *A. phagocytophilum* P44, or ß-actin. **(C)** Two different derlin-1 antibodies produce comparable immunolabeling patterns of the host cell ER and intravacuolar *A. phagocytophilum* organisms. *A. phagocytophilum* and/or uninfected RF/6A cells were screened with derlin-1 antibodies obtained from two different commercial sources. Scale bars, 5 μm. Results shown are representative of two experiments with similar results.

To ensure that derlin-1 immunolabeling of intravacuolar *A. phagocytophilum* organisms was not due to cross-reactivity of the antibody with a bacterial protein, anti-derlin-1 was used to probe Western blotted lysates of uninfected HL-60 cells and host cell-free *A. phagocytophilum* bacteria. A single band of the expected size for derlin-1 (22 kDa) was detected for the HL-60 cell sample, while no band was detected for the *A. phagocytophilum* sample (Figure 3A). Stripping and reprobing the blot with ß-actin and P44 antibodies confirmed sample purity. As a complementary approach, derlin-1 expression was knocked down in HEK-293T cells using siRNA. HEK-293T cells were necessary for this purpose because they not only support *A. phagocytophilum* infection, but also are highly amenable to transfection, whereas RF/6A and HL-60 cells are not (Niu et al., 2012; Beyer et al., 2014; Truchan et al., 2016). HEK-293T cells treated with derlin-1-targeting or non-targeting siRNA were infected with *A. phagocytophilum* followed by Western blot analysis using derlin-1 antibody at 24 h post-infection. Uninfected HEK-293T cells were included as a control. If derlin-1 antibody non-specifically recognized an *A. phagocytophilum* protein, then knocking down host cell derlin-1 would have no effect on the antibody's ability to recognize the cross-reactive bacterial protein. As observed for HL-60 cells, the antibody detected a single band of the expected size for derlin-1 in all three samples. The band of interest was in considerably lower abundance in the infected, derlin-1 siRNA treated sample (Figure 3B). To verify that the ability to immunolabel *A. phagocytophilum* organisms was not unique to the derlin-1 antibody that had been used for all experiments performed thus far, indirect immunofluorescence analyses of *A. phagocytophilum* infected RF/6A cells were repeated to compare this antibody alongside a second derlin-1 antibody purchased from a different company. The immunolabeling patterns of the host cell ER and intravacuolar bacteria were comparable for both antibodies (Figure 3C). Taken together, these data confirm that derlin-1 antibody labeling of *A. phagocytophilum* organisms within the ApV is not due to cross-reactivity and is reproducible for two separate derlin-1 antibodies.

### Ectopically expressed mCherry-derlin-1 is delivered into the ApV, but inhibits its development

**Figure 4:**
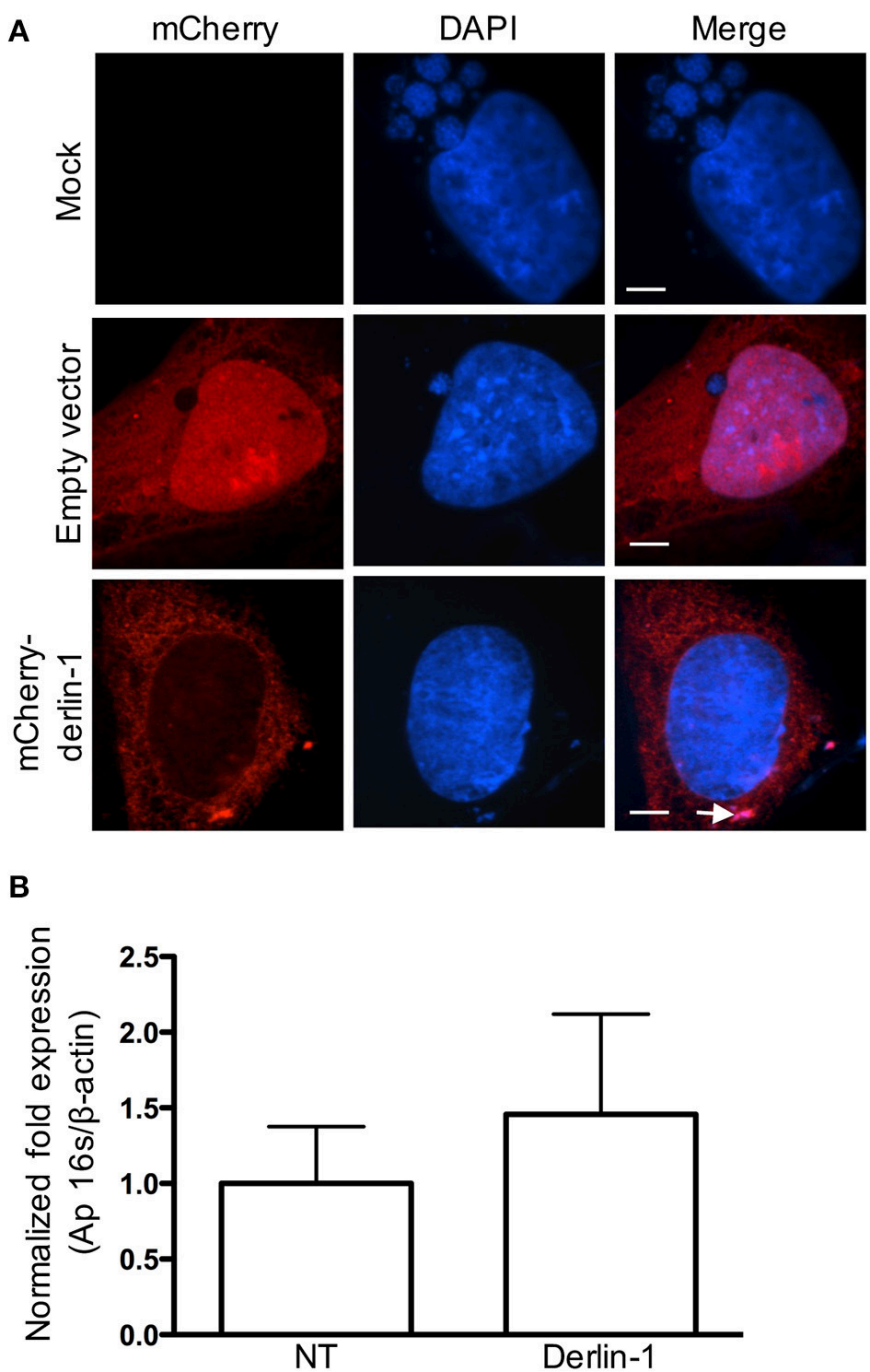
**Ectopically overexpressed mCherry-derlin-1 is delivered into and inhibits development of the ApV, and knocking down derlin-1 increases the *A. phagocytophilum* bacterial load. (A)** mCherry-derlin-1 is delivered into the ApV, associated with intravacuolar *A. phagocytophilum* bacteria, and inhibits ApV development. HEK-293T cells transfected to express mCherry, mCherry-derlin-1, or mock-transfected were incubated with *A. phagocytophilum*. At 24 h, the cells were fixed, stained with DAPI, and visualized using LSCM. The white arrow in **(A)** denotes a small mCherry-derlin-1-positive ApV. Scale bars, 0.5 μm. **(B)** The *A. phagocytophilum* load is increased in derlin-1 siRNA-treated cells. HEK-293T cells were treated with derlin-1-targeting or non-targeting (NT) siRNA for 72 h. Following siRNA treatment, the cells were infected with *A. phagocytophilum* for 24 h and total DNA was isolated and subjected to QPCR analysis.

As an additional means of confirming that derlin-1 associates with intravacuolar *A. phagocytophilum* organisms, HEK-293T cells were transfected to express mCherry-tagged derlin-1 or mCherry alone and subsequently infected. At 24 h, mCherry was diffusely distributed throughout the cytosol and nuclei of transfected cells, but not in the ApV lumen (Figure 4A). mCherry-derlin-1 signal exhibited a reticulate-like pattern reminiscent of the ER, as previously described (Nery et al., 2011), and also labeled DAPI-stained intravacuolar *A. phagocytophilum* bacteria. Notably, however, ApVs were considerably smaller both in diameter and in number in cells expressing mCherryl-derlin-1 as compared to cells expressing only mCherry or mock-infected cells. This result was consistent whether the cells were transfected prior to or after infection. Thus, fluorescently-tagged overexpressed derlin-1 is delivered into the ApV lumen and associates with *A. phagocytophilum* bacteria. However, ApV development is pronouncedly hampered in these cells. We rationalized that derlin-1 may become detrimental to *A. phagocytophilum* at higher levels. We further hypothesized that if derlin-1 negatively influences the bacterium, then knocking down derlin-1 levels would lead to higher *A. phagocytophilum* loads. Indeed, QPCR analyses of HEK-293T cells that had been treated with derlin-1 or non-targeting siRNA prior to infection confirmed that the *A. phagocytophilum* load was higher, albeit insignificantly, in cells in which derlin-1 had been knocked down (Figure 4B). This result indirectly suggests that derlin-1 and cellular processes that it regulates may be detrimental to *A. phagocytophilum* intracellular growth.

### *A. marginale*-occupied vacuoles (AmVs) engage the ER

**Figure 5:**
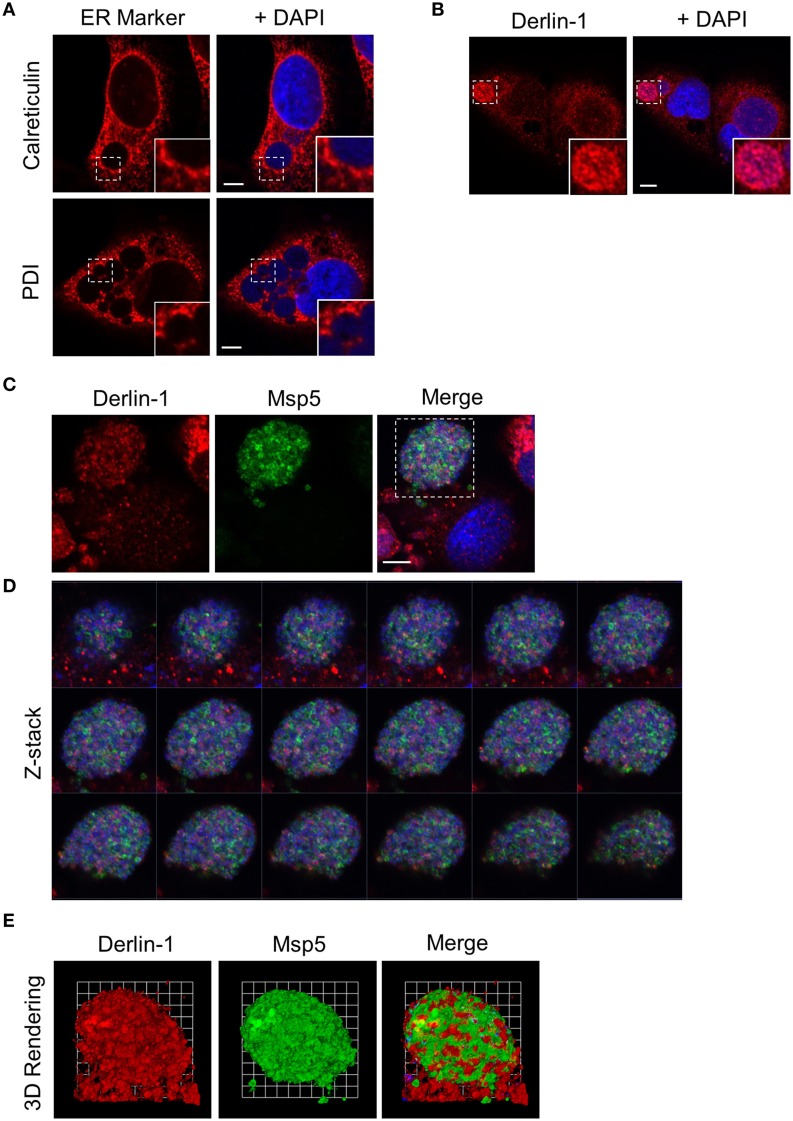
**AmVs interact with the ER in mammalian host cells. (A,B)** The AmV interacts with the ER and ER-derived vesicles are delivered into its lumen. *A. marginale* infected RF/6A cells were screened with antibodies against calreticulin **(A)**, PDI **(A)**, or derlin-1 **(B)**. The regions that are demarcated by hatched lined boxes indicate the regions magnified in the insets that are demarcated by solid lined boxes. **(C–E)** Derlin-1-positive vesicles are present within the AmV in close association with *A. marginale* bacteria. *A. marginale* infected RF/6A cells were screened with antibodies against derlin-1 and Msp5 and visualized using LSCM. **(D)** Z-stack series shows derlin-1-labeled vesicles within the AmV. Successive focal planes of the region in **(C)** that is demarcated by a hatched line box are presented. **(E)** 3D rendering of the Z-stack series presented in **(D)** shows derlin-1-positive vesicles in close proximity to intravacuolar *A. marginale* organisms. Host cell nuclei and bacteria were stained blue with DAPI. Scale bars, 5 μm. Results shown are representative of two experiments with similar results.

To determine if the ability to interface with the ER is conserved in the genus *Anaplasma*, the above analyses were extended to *A. marginale* infected RF/6A cells. Calreticulin and PDI pronouncedly accumulated at the peripheries of AmVs (Figure 5A). Derlin-1-positive vesicles were detected around and within AmV lumen in close proximity *A. marginale* organisms (Figures 5B,C). Z-stack analysis and 3D rendering of a representative AmV from infected cells that had been stained with DAPI and labeled with antibodies against derlin-1 and the *A. marginale* OMP, Msp5 (Visser et al., 1992), confirmed delivery of ER-derived vesicles into the AmV and their association with the bacteria (Figures 5C–E; Supplementary Movie 2). Thus, identical to that observed for the ApV, the AmV interacts with the ER in mammalian host cells and ER derived vesicles are translocated into its lumen.

### *Anaplasma* spp.-occupied vacuoles interact with both the RER and SER, and RER derived vesicles are delivered into the ApV lumen

**Figure 6:**
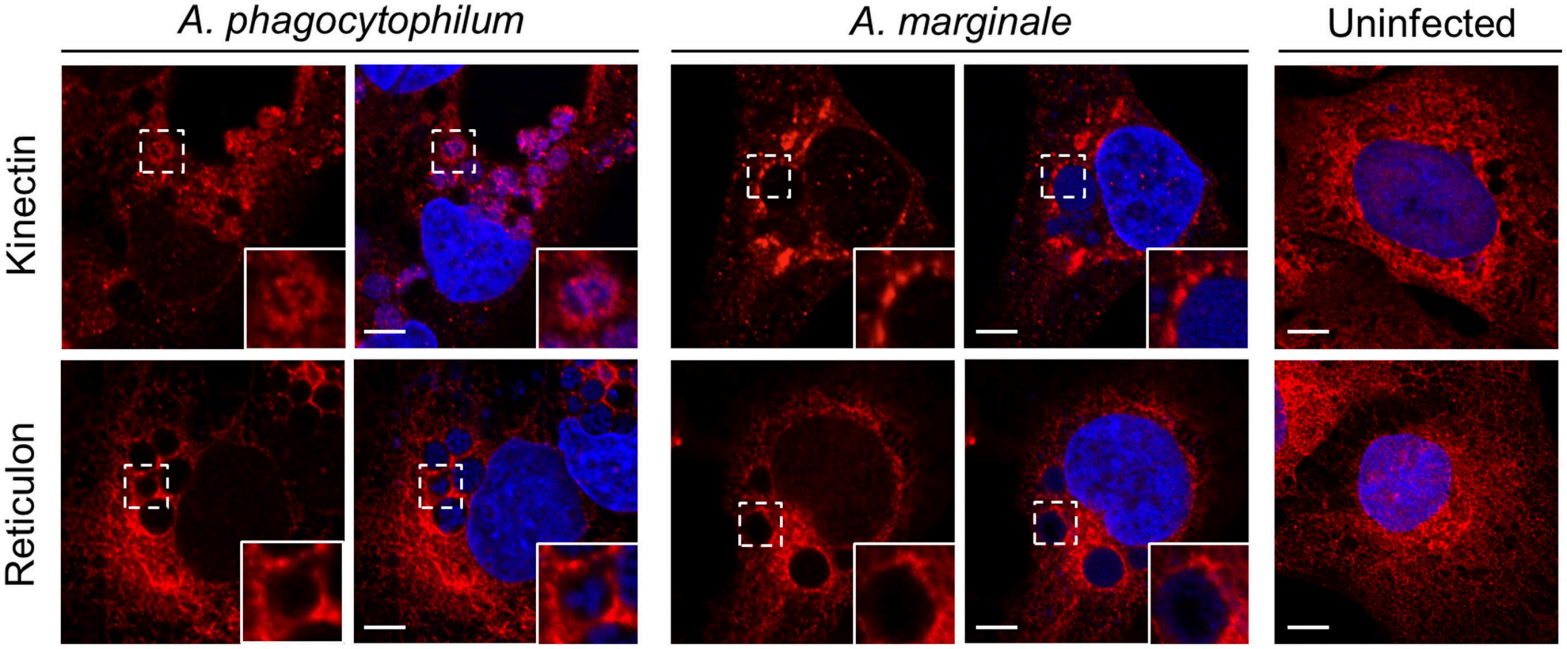
**ApVs and AmVs interact with both the RER and SER**. Uninfected, *A. phagocytophilum* infected, or *A. marginale* infected RF/6A cells were screened with antibodies targeting kinectin-1 or reticulon-4, which are markers for the RER and SER, respectively, and examined using LSCM. Host nuclei and bacterial DNA were stained with DAPI. Scale bars, 5 μm. Results shown are representative of two experiments with similar results.

Because antibodies specific for markers of both the RER and SER were used in the above analyses, it was unclear if the ApV and AmV engage one or both of these compartments. Therefore, RF/6A cells infected with either bacterium were screened with antibodies against kinectin-1 or reticulon-4, which are found on the membranes of the RER and SER, respectively (Lynes and Simmen, 2011; Terasaki et al., 2013). Both markers pronouncedly localized in aggregate patterns around ApVs and AmVs (Figure 6). Vesicles positive for kinectin-1, but not reticulon-4 were detected within the ApV in close apposition to DAPI-stained *A. phagocytophilum* organisms. Neither kinectin-1 nor reticulon-4 were detected within the AmV. Thus, the ApV and AmV associate with both the SER and RER, but only RER-derived vesicles are translocated into the ApV lumen.

### Structured illumination microscopy analyses confirm that ER derived vesicles are present in *Anaplasma* spp.-occupied vacuoles and associate with intravacuolar bacteria

**Figure 7:**
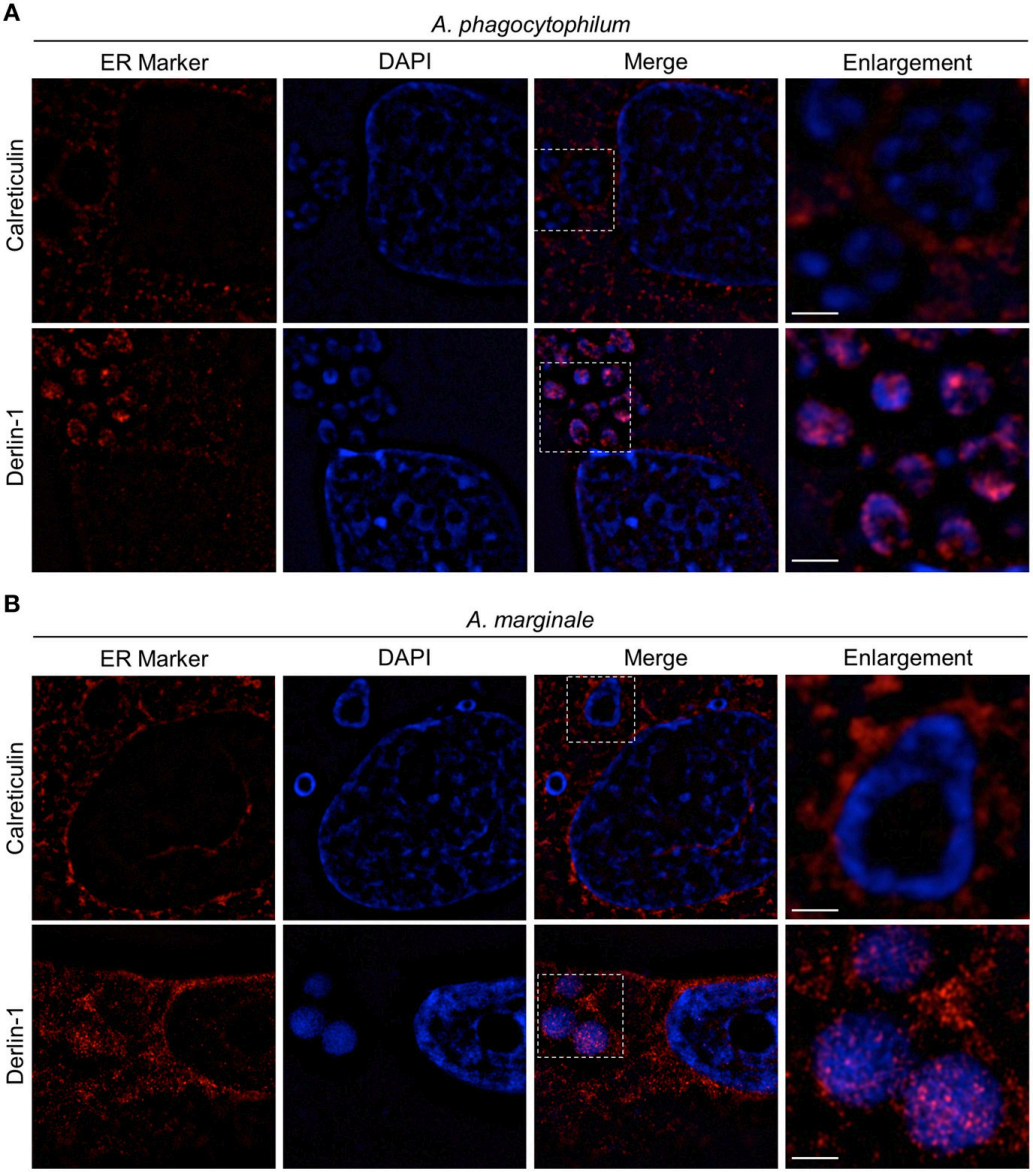
**SIM analyses confirm that calreticulin- and derlin-1-positive vesicles are present in the ApV and AmV lumen in close proximity to intravacuolar *Anaplasma* spp. bacteria**. *A. phagocytophilum* **(A)** and *A. marginale* infected RF/6A cells **(B)** that had been screened with antibodies against calreticulin or derlin-1 were examined by SIM. The regions in the Merge panels that are demarcated by hatched line boxes indicate the regions that are magnified in the Enlargement panels. Host cell nuclei and bacterial DNA were stained with DAPI (blue). Scale bars, 5 μm. Results shown are representative of two experiments with similar results.

LSCM analyses suggested that ER derived vesicles are present within the ApV and AmV intravacuolar bacteria. However, because LSCM has a lateral resolution of approximately 200 nm, structures that are separated by less than 200 nm in the XY plane appear as single fused objects (Allen et al., 2014). Within cells, and presumably within POVs, macro-molecular associations occur in spatial distances of less than 200 nm. To more accurately evaluate if ER derived vesicles associate with intravacuolar *Anaplasma* spp. organisms, structured illumination microscopy (SIM), a form of super-resolution microscopy that has a lateral resolution of approximately 100 nm (Allen et al., 2014), was employed. Similar to that observed via LSCM, SIM imaging detected signals for calreticulin and derlin-1 as vesicle-like patterns that accumulated around the peripheries of clusters of DAPI-stained *A. phagocytophilum* and *A. marginale* bacteria (Figure 7), which were presumably within POVs. Moreover, derlin-1 signal also pronouncedly colocalized with bacteria-associated DAPI signals. These data verify that derlin-1-positive ER derived vesicles are within 100 nm of the surfaces of *A. phagocytophilum* and *A. marginale* organisms within POVs.

### *A. phagocytophilum*- and *A. marginale*-occupied vacuoles interact with the ER in tick cells

**Figure 8:**
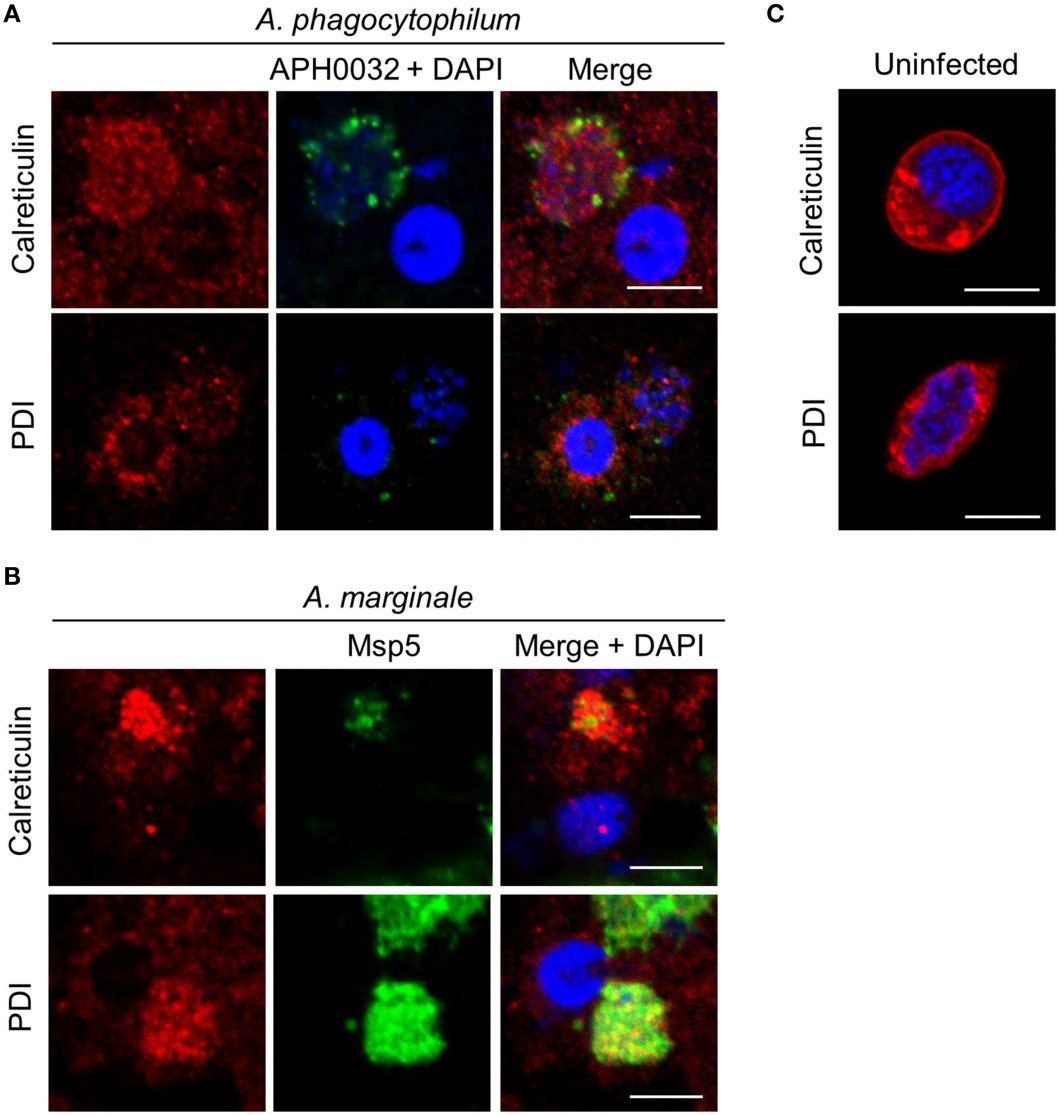
**ApVs and AmVs associate with the ER in ISE6 tick cells**. Uninfected **(C)**, *A. phagocytophilium*- **(A)**, or *A. marginale*-infected ISE6 tick cells **(B)** were labeled with antibodies against either calreticulin or PDI in combination with APH0032 (for *A. phagocytophilum*) or Msp2 (for *A. marginale*) and examined using LSCM. Host nuclei and bacteria were stained blue with DAPI. Scale bars, 5 μm. Results shown are representative of two experiments with similar results.

Because *A. marginale* and *A. phagocytophilum* are tick-transmitted pathogens, it was examined if their POVs interact with the ER in ISE6 tick cells. There is a paucity of commercial antibodies against *I. scapularis* proteins. A BLAST (basic local alignment tool) search revealed that the linear epitopes of human calreticulin and PDI recognized by the commercial antibodies used in this study are present in the *I. scapularis* orthologs of these proteins (data not shown). Uninfected and *A. phagocytophilum*- or *A. marginale*-infected ISE6 cells were subjected to LSCM analyses using these antibodies together with antibody specific for APH0032 (for *A. phagocytophilum*) or Msp5 (for *A. marginale*). Both ER markers exhibited the expected perinuclear and network-like pattern in uninfected cells (Figure 8C). In infected cells, calreticulin- and PDI-labeled vesicles were pronouncedly enriched around ApVs and AmVs and also colocalized with *Anaplasma* organisms within both POV types (Figures 8A,B). Thus, *A. phagocytophilum* and *A. marginale* establish interactions with the ER in not only vertebrate, but also invertebrate host cells.

### Ultrastructural analyses of *A. phagocytophilum*- and *A. marginale*-occupied vacuole interactions with the ER

**Figure 9:**
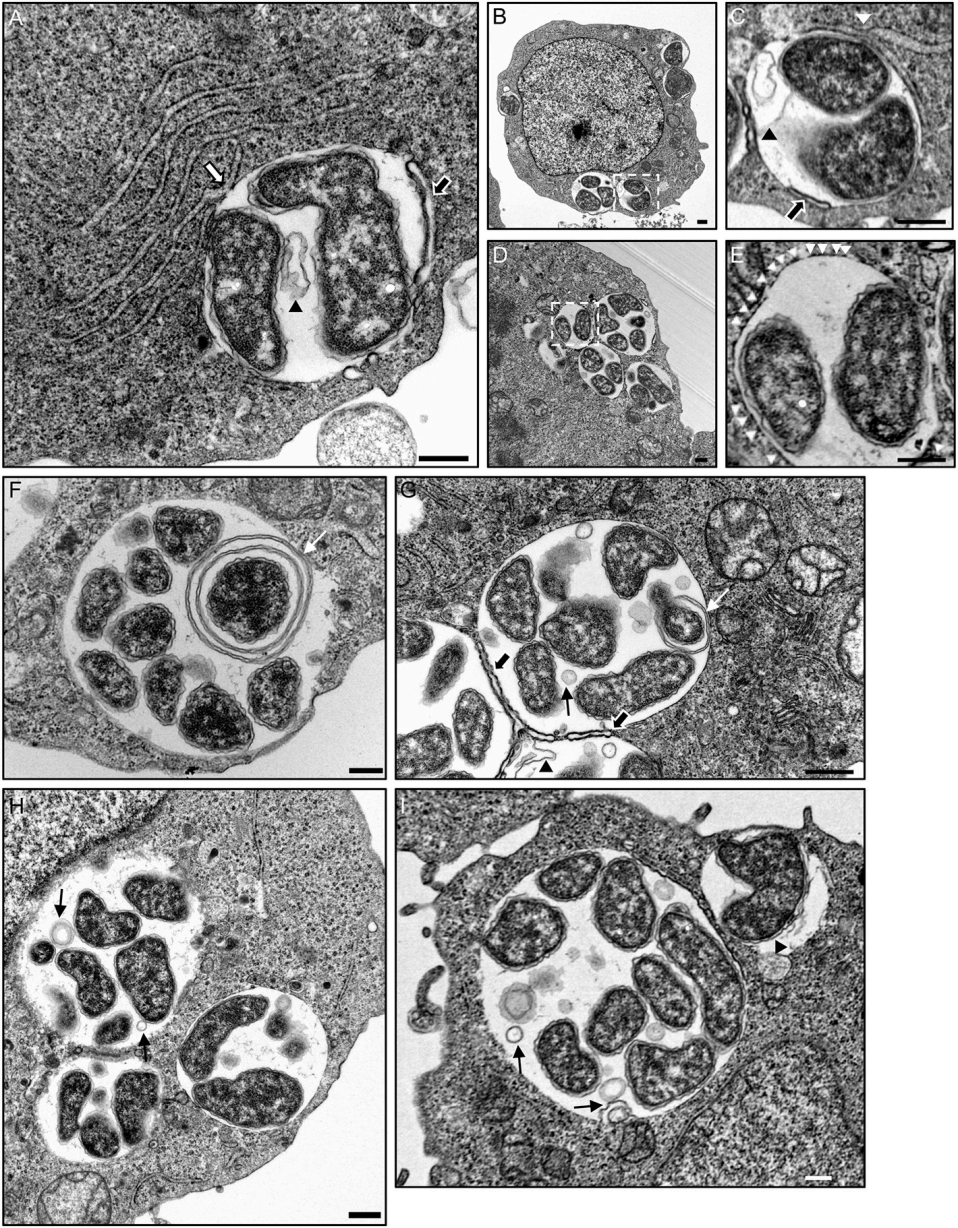
**The ApV interacts with the ER as visualized by TEM**. *A. phagocytophilum* infected HL-60 cells were examined by TEM. White hatched boxes denote areas in the images presented in **(B,D)** that are presented as enlarged panels in **(C,E)**, respectively. The white arrow in **(A)** denotes a RER-ApV contact site. Black arrows in **(A,C,G)** denote autophagosomes contacting the ApV membrane. Black arrowheads in **(A,C,G)**, and **(I)** demarcate autophagic bodies present within the ApV lumen. White arrowheads in **(E)** denote ribosomes that label the cytosolic face of the ApV membrane. Thin white arrows in **(F,G)** demarcate membranes within the ApV lumen associating with *A. phagocytophilum* organisms. Thin black arrows in **(G,H,I)** point to vesicles within the ApV lumen in close apposition to *A. phagocytophilum* bacteria. Scale bars, 0.5 μm. Results shown are representative of two experiments in which a combined total of over 200 different electron micrographs were analyzed.

**Figure 10:**
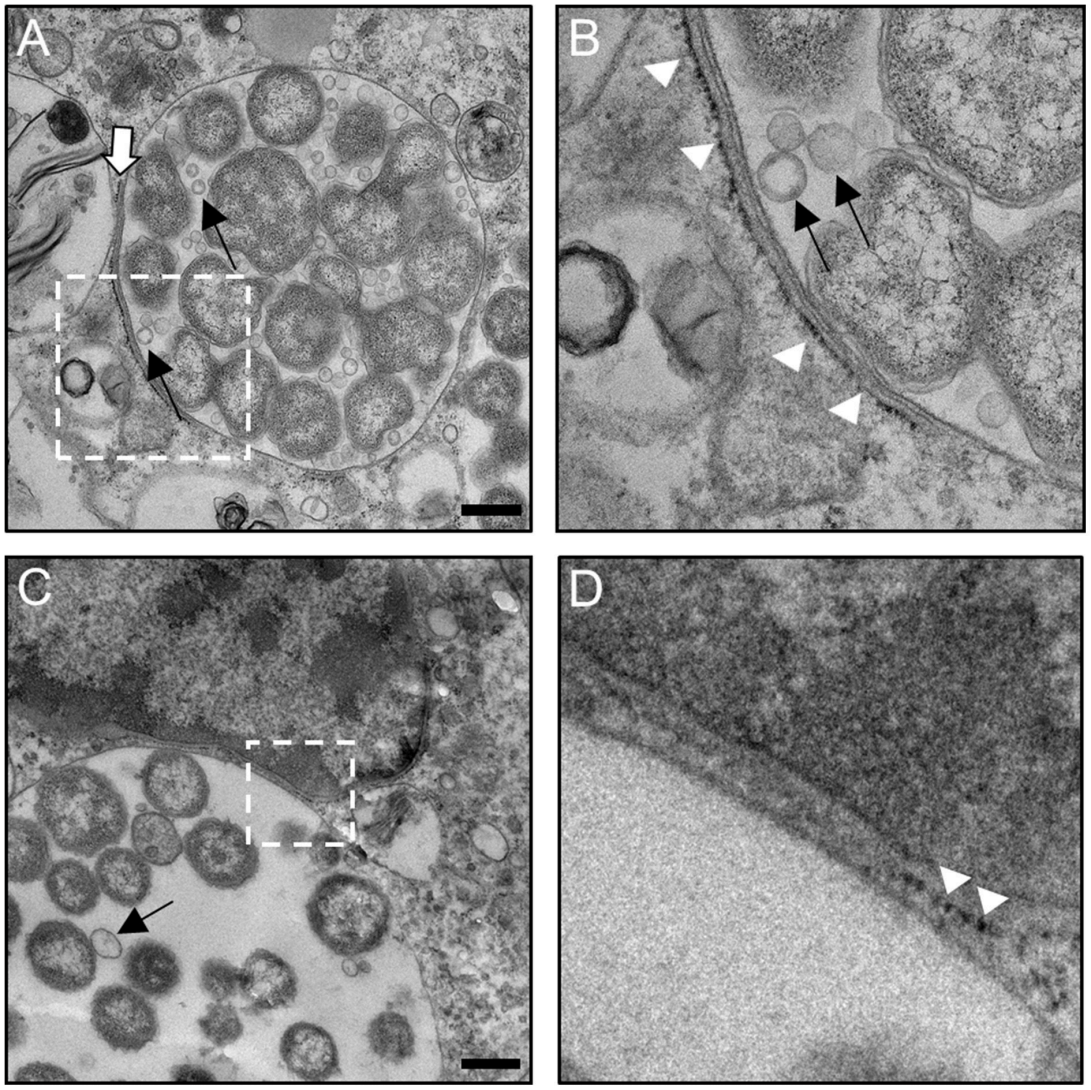
**The AmV interacts with the ER as visualized by TEM**. *A. marginale* infected ISE6 cells were examined by TEM. White hatched boxes denote areas in the images presented in **(A,C)** that are presented as enlarged panels in **(B,D)**, respectively. The white arrow in **(A)** denotes a RER-AmV contact site. White arrowheads in **(B,D)** point to ribosomes that label the cytosolic face of the AmV membrane. Thin black arrows in **(A–C)** demarcate vesicles within the AmV lumen in close apposition to *A. marginale* organisms. Scale bars, 0.5 μm. Results shown are representative of two experiments in which a combined total of over 40 different electron micrographs were analyzed.

To investigate the interactions of *Anaplasma* spp.-occupied vacuoles with the ER at the ultrastructural level, host cells infected with either species were examined by transmission electron microscopy. As previously reported (Niu et al., 2012), autophagosomes were observed closely apposed to ApVs and autophagic bodies were observed within ApVs in *A. phagocytophilum* infected HL-60 cells (Figures 9A–C,G,I). ApVs (Figure 9A) and AmVs (Figures 10A–D) were observed in close proximity to ribosome studded RER sheets and the RER formed contacts with and/or wrapped portions of both POV types (Figures 9A–C, 10A–D). Sites where the RER formed contacts on the POV membrane's cytosolic face corresponded with where RCs were closely apposed to the POV membrane's lumenal face (Figures 9A,C, 10A–D). Membrane bound vesicles were commonly observed within ApV and AmV lumen in close proximity to bacteria (Figures 9G–I, 10A–C). *A. phagocytophilum* organisms were occasionally observed wrapped by membranes (Figures 9F,G). The cytosolic faces of ApVs (Figures 9D,E) and AmVs (Figures 10A–D) were often studded with ribosomes. While it could not be ruled out that these were cytosolic ribosomes, the data presented thus far suggested that they were likely derived from the RER and possibly signify ER-*Anaplasma* spp.-occupied vacuole membrane fusion. Alternatively, the inability to detect a RER sheet in such instances could simply be due to the thin section nature of the sample. In both *A. phagocytophilum* infected HL-60 cells (Figure 9B) and *A. marginale* infected ISE6 cells (Figures 10C,D), POVs were commonly observed butted up against the nucleus, phenomena that are potentially related to these pathogens' ER tropism, given that the ER membrane is continuous with the nuclear membrane (Puhka et al., 2012). These data substantiate the association of the ER with *A. phagocytophilum*- and *A. marginale*-occupied vacuoles, suggest potential fusion of and/or synapse formation between POV and ER membranes, and evidence the presence of vesicles and membranes within POVs in close proximity to bacteria.

### Association of the ApV with the ER is Rab10-independent

**Figure 11:**
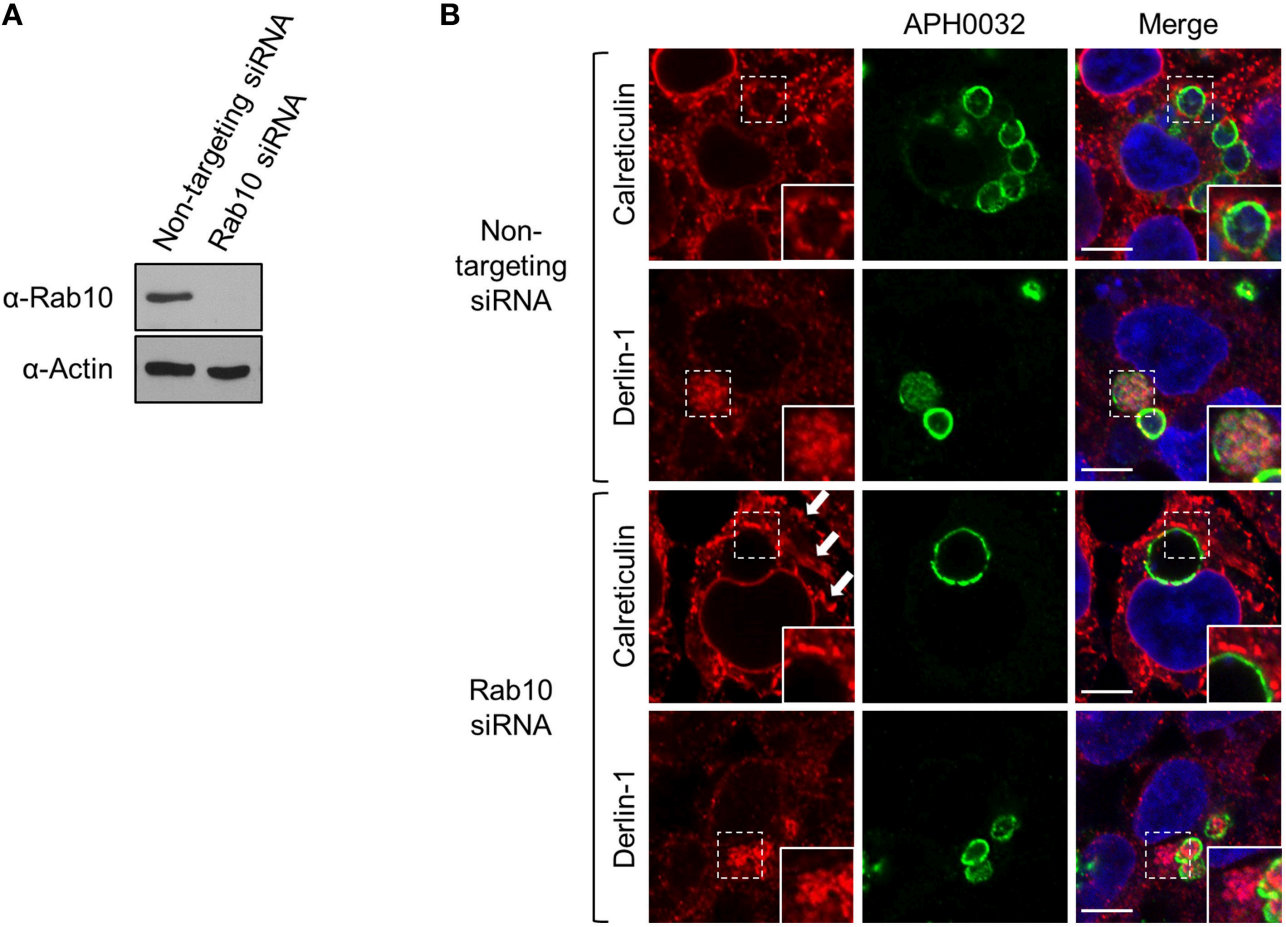
**ApV association with the ER is Rab10-independent**. HEK-293T cells were treated with Rab10-targeting or non-targeting siRNA for 72 h. **(A)** Lysates of non-targeting or Rab10 siRNA treated cells were examined by Western blot for Rab10 knockdown. **(B)** Following siRNA treatment, the cells were infected with *A. phagocytophilum* for 48 h, fixed, screened with antibodies against APH0032 and calreticulin or derlin-1, and examined using LSCM. Host cell nuclei and bacterial DNA were stained with DAPI. Regions demarcated by hatched line boxes are magnified in the corresponding inset images that are denoted by a solid line boxes. Arrows point to regions of expansive cisternae, which is characteristic ER morphology in Rab10-depleted cells. Scale bars, 5 μm. Results shown are representative of two experiments with similar results.

Among the Rab GTPases that *A. phagocytophilum* selectively recruits to its vacuole are Rab10 and Rab1, both of which are ER associated (Stenmark, 2009; Huang et al., 2010a; English and Voeltz, 2013). Whereas, Rab10 is abundantly detected on ApVs, Rab1 is considerably less so (Huang et al., 2010a). To determine if the association between the ApV and ER is Rab10-dependent, ER recruitment to the ApV was assessed in HEK-293T cells that had been treated with Rab10 or non-targeting siRNA. Rab10 knockdown was confirmed by Western blot (Figure 11A). Despite multiple attempts, Rab1 could not be knocked down. As previously reported (English and Voeltz, 2013), Rab10-depletion resulted in altered ER morphology. The organelle had lost its network appearance and instead displayed regions of expansive cisternae (Figure 11B). No difference in calreticulin accumulation at the ApV periphery or the abundant detection of derlin-1 within the ApV lumen was observed between Rab10 knockdown and control cells (Figure 11B). Thus, *A. phagocytophilum* does not require Rab10 to establish interactions with the ER or to facilitate translocation of ER-derived vesicles into its vacuole.

## Discussion

In this study, we demonstrated that the ApV and AmV engage the ER and that ER derived vesicles are delivered into their lumen in mammalian and tick host cells. This strategy is likely important to *Anaplasma* spp. intracellular survival, as it is initiated in the early hours following the bacterial entry event that generates the POV and continues throughout the entirety of the infection cycle. The delivered vesicles are positive for derlin-1, an ER membrane-associated protein that pronouncedly localizes to the ERQC dynamically and on demand during ER stress (Lederkremer, 2009; Groisman et al., 2011; Leitman et al., 2014; Benyair et al., 2015). This suggests that the bacteria may selectively hijack membrane traffic from the ERQC. Rab10 is important for vesicular trafficking from the TGN, for ER membrane dynamics, and for maintaining ER morphology, but has not been implicated in ERQC formation or function (Liu and Storrie, 2012; English and Voeltz, 2013). Consistent with this, while Rab10 is essential for *A. phagocytophilum* TGN parasitism (Truchan et al., 2016), it is dispensable for it to interface with the ER. Thus, the bacterium targets the TGN and ER by distinct mechanisms. *Legionella pneumophila* modulation of Rab1 is essential for the bacterium to pirate membrane traffic emanating from ERESs (Sherwood and Roy, 2013). Though our inability to knock down Rab1 prevented us from discerning if Rab1 is important for *A. phagocytophilum* to hijack ER membrane traffic, we speculate that it is not, primarily because, like Rab10, Rab1 does not associate with the ERQC (Lederkremer, 2009). Whereas, *Anaplasma* spp.-occupied vacuoles associate with the RER and SER, RER derived vesicles were detected within lumen of ApVs but not AmVs. Thus, while targeting the ER is a conserved thematic strategy between *A. phagocytophilum* and *A. marginale*, species-specific differences exist.

Consistent with immunolabeling of endogenous derlin-1, mCherry-tagged derlin-1 is detected within the ApV lumen and appears to label intravacuolar bacteria. However, ApVs are pronouncedly smaller in both size and number in cells ectopically overexpressing mCherry-derlin-1, but not mCherry. This result suggests that overstimulation of a derlin-1-influenced cellular process may result in aberrantly small ApVs. Derlin-1 overexpression has been reported to lead to severe ER stress and ER stress-induced apoptosis (Liang et al., 2014), either or both of which may prevent *A. phagocytophilum* intracellular growth. A recent study indicated that the translocation of ER associated markers may be exaggerated during chemical fixation and argued that live cell imaging is the gold standard for assessing the significance of such phenomena (Kokes and Valdivia, 2015). Due to the toxic effect of mCherry-derlin-1 overexpression, we could not extend our analyses using live cell imaging. That being said, several lines of indirect evidence collectively argue that derlin-1-positive vesicles are delivered into the ApV lumen to associate with intravacuolar bacteria. First, antibodies specific for derlin-1, but neither calreticulin nor PDI immunolabeled intravacuolar *A. phagocytophilum* organisms. Second, derlin-1 antibody specifically recognizes its target protein in eukaryotic cells and does not cross-react with an *A. phagocytophilum* protein. Third, two different derlin-1 antibodies immunolabeled the bacteria. Fourth, mCherry-derlin-1, but not mCherry is detected within the ApV lumen even though both proteins are abundantly present and adjacent to the ApV membrane in transfected cells. Thus, while live cell imaging is a powerful and important technique for assessing the delivery of organelle-derived vesicular traffic into POV lumen and should be employed whenever possible, it is not always going to be a feasible option. In such instances, employing multiple alternative control experiments, as we did herein, can circumvent this technical obstacle to reach a valid conclusion.

*Anaplasma* spp.-occupied vacuoles contact the ER and possibly fuse with its membrane at distinct sites. *Anaplasma* bacteria associate closely on the lumenal sides of such POV-ER membrane contact sites, an observation that is reminiscent of the pathogen synapses formed by chlamydial Type III secretion system apparatuses that connect intravacuolar chlamydiae to the inclusion membrane at ER contact sites (Dumoux et al., 2012). Such chlamydial inclusion membrane-ER points of contact are also where the bacterial inclusion membrane protein, IncD, recruits host ceramide transfer protein (CERT) that, in turn, interacts with its binding partners and ER membrane resident proteins, VAMP-associated protein-A/B. Recruitment of host sphingomyelin synthases 1 and 2 together with CERT completes a proposed sphingomyelin biosynthetic factory at this host-pathogen interface that may be important in satisfying the chlamydial need for sphingomyelin and may also be a pathogen-orchestrated signaling platform (Derré et al., 2011; Elwell et al., 2011; Agaisse and Derre, 2014). Given that we recently demonstrated that host cell-free *A. phagocytophilum* DCs are enriched in multiple ceramide and sphingomyelin sub-species (Truchan et al., 2016), moving forward it will be important to discern whether *Anaplasma* spp.-occupied vacuoles have similar metabolic/signaling platforms at their ER-POV membrane contact sites, the bacterial factors responsible for facilitating their formation, and their contributions to *Anaplasma* spp. pathobiology.

Though other intracellular bacterial pathogens have been shown to replicate within ER-derived compartments (Swanson and Isberg, 1995; Roy et al., 2006; Dumoux et al., 2012; Celli and Tsolis, 2015), the concept of intact ER vesicles being delivered into the POV is fairly novel. Prior to this study, the chlamydial inclusion was the only POV demonstrated to intimately contact the ER and ingest intact ER derived vesicles into its lumen (Dumoux et al., 2012). The ApV and the chlamydial inclusion each also associates with the Golgi apparatus and selectively hijacks its exocytosed vesicles, which, like ER vesicles, are translocated into the POV lumen to associate with intravacuolar bacteria (Heuer et al., 2009; Capmany and Damiani, 2010; Moore et al., 2011; Pokrovskaya et al., 2012; Al-Zeer et al., 2014; Dille et al., 2014; Truchan et al., 2016). Hijacking ER- and Golgi-derived traffic is essential for chlamydiae to convert from the replicative to infectious form (Heuer et al., 2009; Dumoux et al., 2012). TGN parasitism is critical for *A. phagocytophilum* to complete its biphasic developmental cycle (Dumoux et al., 2012; Truchan et al., 2016). Whether pirating a derlin-1-positive ER sub-compartment contributes to *Anaplasma* spp. RC-to-DC conversion is unknown, as is the pathobiological benefit that hijacking this specific ER domain affords. Regardless, data presented here and in our previous study demonstrate the importance of hijacking multiple secretory organelles to *Anaplasma* spp. infection. Moreover, this study adds to a growing body of literature that demonstrates that pathogens in the families *Anaplasmataceae* and *Chlamydiaceae*, while evolutionarily distinct in terms of whether or not they are vector-transmitted and the host cell types that they infect, exhibit a conserved demand for parasitizing the secretory pathway and targeting secretory organelle derived vesicles into their POVs (Beatty, 2006; Cocchiaro et al., 2008; Capmany and Damiani, 2010; Dumoux et al., 2012; Boncompain et al., 2014; Truchan et al., 2016). While the responsible mechanism(s) for such membrane fusion-independent delivery is undefined, it was recently demonstrated that two *Toxoplasma gondii* encoded proteins that localize to the parasitophorous vacuole membrane form a conduit that facilitates passage of small molecules between the vacuole and host cytosol (Gold et al., 2015). Perhaps functionally analogous proteins exist on bacterial POVs to import intact host cell derived vesicles.

What possible benefits could hijacking ER traffic afford *A. phagocytophilum* and *A. marginale*? As the pathogens are auxotrophic for most amino acids (Brayton et al., 2005; Rikihisa, 2011), ER vesicles transported into their vacuoles could provide proteins that get degraded and used as an amino acid source, as has been demonstrated for autophagic bodies that are delivered into the ApV as a result of ApV-autophagosome fusion (Niu et al., 2012). *Anaplasma* spp. could use the amino acids for protein synthesis or as a source of carbon, nitrogen, and energy by feeding the amino acids into the Kreb's cycle. The latter strategy has been well-documented for *L. pneumophila* (Pine et al., 1979; Tesh et al., 1983; Price et al., 2014). Autophagosomes are believed to be formed from ER membrane and it was recently found that 70% of autophagosomes contain portions of the ER (Hayashi-Nishino et al., 2009; Shibutani and Yoshimori, 2014). However, autophagosomes do not contain ribosomes, which were detected on or in close apposition to the cytosolic faces of ApVs and AmVs, or calreticulin and PDI (Dunn, 1994; Hayashi-Nishino et al., 2009; Lamb et al., 2013; Shibutani and Yoshimori, 2014), which heavily label *Anaplasma* spp.-occupied vacuoles. Thus, while the ApV engages autophagosomes (Niu et al., 2012), both it and the AmV establish separate interactions with the ER.

The recently penned term, nutritional virulence, refers to the condition that without access to essential nutrients, pathogens cannot survive to cause disease (Abu Kwaik and Bumann, 2013). ER-POV interactions may help satisfy the nutritional virulence requirements of *A. phagocytophilum* during infection of leukocytes and of both it and *A. marginale* during infection of endothelial and tick cells. It will be important to verify which specific ER subdomain(s) *Anaplasma* spp. target, the relevance of this strategy to their infection cycles, and the molecular mechanisms by which they are orchestrated. Such knowledge could potentially be exploited as a novel means for treating human or animal infections caused by *Anaplasma* spp. or for eliminating the pathogens from their tick vectors.

## Author contributions

JC, HT, SN designed experiments and analyzed the data. HT, CC, KH, FM performed the experiments and analyzed the data. JC and HT wrote the paper.

## Funding

This study was supported by funding from National Institutes of Health Grants R01 AI072683, National Center for Advancing Translational Sciences Grant UL1TR000058, and the Center for Clinical and Translational Research Endowment Fund of VCU (to JC). LSCM, SIM, and electron microscopy were performed at the VCU Microscopy Facility, which is supported in part with funding from NIH-NINDS Center core grant 5P30NS047463 and NIH-NCI Cancer Center Support Grant (P30 CA016059). SN: U. S. Department of Agriculture-Agricultural Research Service Project #5348-32000-033-00D and National Institutes of Health R37 AI44005.

### Conflict of interest statement

The authors declare that the research was conducted in the absence of any commercial or financial relationships that could be construed as a potential conflict of interest.
