# Supplementary material for: The Pathogen-Occupied Vacuoles of Anaplasma phagocytophilum and Anaplasma marginale Interact with the Endoplasmic Reticulum
Source: Front Cell Infect Microbiol. 2016 Mar 1;6:22. doi: 10.3389/fcimb.2016.00022 (PMC4771727; doi:10.3389/fcimb.2016.00022)
Supplement: Supplementary file 3 [file DataSheet1.DOCX]

Supplementary Material

The pathogen-occupied vacuoles of *Anaplasma phagocytophilum* and *Anaplasma marginale* interact with the endoplasmic reticulum

Hilary K. Truchan^1,3^, Kathryn S. Hebert^1^, Chelsea L. Cockburn^1^, Forgivemore Magunda^2^, Susan M. Noh^2^, Jason A. Carlyon^1^*

^1^Department of Microbiology and Immunology, Virginia Commonwealth University School of Medicine, Richmond, Virginia, USA

^2^Department of Veterinary Microbiology and Pathology, Washington State University, Pullman, Washington, USA

*** Correspondence:** Corresponding Author: jason.carlyon@vcuhealth.org

# Supplementary Data

**Supplementary Movie 1: Derlin-1-positive ER vesicles are delivered into the ApV lumen where they associate with *A. phagocytophilum* organisms.** *A. phagocytophilum* infected RF/6A cells were screened with antibodies against derlin-1 (red) and APH0032 (green), stained with DAPI (blue), and visualized by LSCM. Z-stack images obtained for a representative ApV in Figure 2B were used for 3D rendering and movie generation.

**Supplementary Movie 2: Derlin-1-positive ER vesicles are delivered into the AmV lumen where they associate with *A. marginale* organisms.** *A. marginale* infected RF/6A cells were screened with antibodies against derlin-1 (red) and Msp5 (green), stained with DAPI (blue), and visualized by LSCM. Z-stack images obtained for a representative AmV in Figure 3C were used for 3D rendering and movie generation.
